# Cost-Effectiveness of a Child and Parent Mindfulness-Based Training Versus Medication for Childhood ADHD

**DOI:** 10.1177/10870547261469623

**Published:** 2026-08-24

**Authors:** Rachel T. van der Meulen, Francisca J. A. van Steensel, Esther I. de Bruin, Wiebe Korf, Susan M. Bögels

**Affiliations:** 1 University of Amsterdam, The Netherlands; 2 UvA Minds, Academic Treatment Center, Amsterdam, The Netherlands

**Keywords:** childhood ADHD, mindfulness, medication, cost-effectiveness

## Abstract

**Objective:**

ADHD imposes substantial societal costs. This randomized controlled trial examined the cost-effectiveness of family mindfulness training (“MYmind”) versus medication in childhood ADHD.

**Method:**

An economic evaluation with a 10-month time horizon was conducted from a societal perspective. Children (*N* = 88, *M *= 11.27 years, 70% boys) with ADHD were randomized to MYmind (8 weekly 1.5-hr group mindfulness sessions for children and parallel for their parents, plus a 2-months follow-up) or medication (4-months immediate-release methylphenidate). During the following 6 months, families had free treatment choice, allowing crossover. Costs were assessed using retrospective cost-questionnaires. Primary outcome measures were children’s inattention and quality-adjusted life years (QALYs). Secondary analyses included: hyperactivity/impulsivity, internalizing and externalizing problems as outcome measures, taking a healthcare (payers) perspective, shorter time horizons (2 and 4 months), a different inattention questionnaire, per protocol analyses, and exclusion of crossover participants.

**Results:**

At 10 months, primary cost-effectiveness analyses indicated that MYmind was inferior to medication for inattention but had higher QALY gains at higher costs. Specifically, MYmind had a 27% probability of being cost-effective for inattention and a 30% probability for QALYs. Secondary analyses showed similar results with even lower chances for mindfulness being cost-effective in the shorter-term horizons. Notably, in the subgroup excluding crossover participants, MYmind was dominant over medication, with an 81% chance to be cost-effective for both outcomes.

**Conclusions:**

MYmind was less cost-effective than medication in the short and medium term. Long-term cost-effectiveness of MYmind remained low for the total group, but was high among participants who did not crossover to the alternative treatment.

## Introduction

Attention deficit hyperactivity disorder (ADHD) is a neurodevelopmental behavioral disorder, affecting an estimated 7.6% of children aged 3 to 11 and 5.6% of adolescents aged 12 to 18 (Salari et al., 2023). ADHD is characterized by behavioral symptoms of inattention and disorganization, and/or hyperactivity-impulsivity (American Psychiatric Association, 2022) and is associated with impairment in multiple areas of children’s lives, including academic performance, social functioning, and overall quality of life (Arnold et al., 2015; Lee et al., 2016; Ros & Graziano, 2017). ADHD co-occurs often with other mental disorders that include both internalizing problems (e.g., anxiety) and externalizing problems (e.g., oppositional behavior; Reale et al., 2017). Comorbidity rates in children with ADHD range from about 40% to 80% depending on the sample, with higher rates in clinically referred children with ADHD (67%–87%; Reale et al., 2017).

ADHD is related to costly negative outcomes in adolescence and adulthood, including higher rates of substance abuse, delinquency, work loss, and traffic accidents among individuals with ADHD (Matza et al., 2005; Tarver et al., 2014). The economic implications of ADHD are substantial and include both higher medical and societal costs compared to individuals without ADHD. A review including all global evidence on the economic burden of ADHD found estimates based on total annual costs ranging from $US 831 to 20,751 for per person estimates, and from $US 356 million to 20.27 billion for national estimates (Chhibber et al., 2021). These costs included direct (medical and non-medical), indirect, and education and justice system costs. Costs tend to increase when comorbid mental health disorders are present (Guevara et al., 2001).

The primary treatments for ADHD include medication and psychosocial interventions, such as school-based behavioral programs and behavioral parent trainings. Although both approaches are effective in reducing ADHD symptoms, medication consistently demonstrates larger effect sizes than psychosocial therapies (Türk et al., 2023). Stimulant medication yields response rates of approximately 80% (Zheng, 2022), and methylphenidate remains the internationally recommended first-line treatment for childhood ADHD according to major clinical guidelines (Türk et al., 2023). Beyond symptom reduction, ADHD treatment is associated with decreased risk of adverse and costly outcomes, including criminality, traffic accidents, occupational impairment, substance use, and obesity (Shaw et al., 2012). However, while existing treatments reduce the negative impact of ADHD on life functioning, they rarely lead to normalization of functioning (Shaw et al., 2012), underscoring the need for continued innovation in treatment development.

Although demonstrating clinical effectiveness is essential, it is also important to determine whether an intervention provides good value for money. Cost-effectiveness analysis evaluates whether the health benefits of a treatment justify the financial resources required to deliver it. In such analyses, both outcomes (e.g., symptom reduction or improvements in quality of life) and costs (e.g., healthcare use, educational support, parental productivity loss) are taken into account. One commonly used outcome in cost-effectiveness research is the quality-adjusted life year (QALY), which combines both the quantity and quality of life into a single measure. One QALY represents 1 year of life in perfect health. Cost-effectiveness studies estimate how much additional cost is required to gain one additional QALY or one additional treatment responder. These estimates are then compared to commonly accepted “willingness-to-pay” thresholds – that is, the amount society is considered willing to pay for a meaningful health improvement – to determine whether an intervention can be considered to be implemented (for further reading see e.g., Ryen & Svensson, 2014).

To date, relatively little research has directly compared the cost-effectiveness of different ADHD treatments. A recent systematic review of economic evaluations, encompassing 29 studies, concluded that stimulant medication is cost-effective for children and adolescents with ADHD (Dijk et al., 2021). In contrast, only a few studies have examined the cost-effectiveness of psychosocial interventions, typically focusing on behavioral parent training or school-based behavioral programs. These studies often employ short time horizons –ranging from 13 weeks to 14 months – which limits the strength of the conclusions that can be drawn. The Multimodal Treatment of ADHD (MTA) study directly compared behavioral management training (targeting parents, children, and teachers) with medication and found that behavioral interventions were generally dominated by medication management in terms of cost-effectiveness (Foster et al., 2006; Jensen et al., 2005). Overall, Dijk et al. (2021) concluded that treating ADHD – whether through medication or behavioral training (primarily parent-focused) – is generally cost-effective compared with no treatment, placebo, or usual care (Dijk et al., 2021).

Although most research concerning (cost-)effectiveness of ADHD treatments focuses on medication, there are increasing concerns about the frequency of methylphenidate prescriptions and its disadvantages (Graham et al., 2010; International Narcotics Control Board, 2015). Side effects of stimulant medication are frequently reported; Charach et al. (2004) report that 68% children with ADHD adhering to medication over a period of 3 years suffer from one or more side effects. Frequently reported side effects include insomnia, loss of appetite, abdominal pain, headache, anxiety, and tics (Storebø et al., 2023). Although rare, serious adverse events have been also reported, for instance psychotic symptoms, arrhythmia, seizures, and hypertension (Storebø et al., 2023). Other concerns of medication are the low treatment adherence; approximately 50% of people with ADHD discontinue medication (Rashid et al., 2017), the fact that symptoms return once medication is discontinued (Taylor et al., 2004), the frequent misuse (Clemow & Walker, 2014), long-term safety (Lange, 2017), and lastly, that ADHD medication becomes less effective in the long run (Taylor, 2019). Therefore, effective non-medical treatments for ADHD are highly needed.

An alternative to medication is the MYmind mindfulness training, designed for children with ADHD and their parents. The MYmind training focuses on providing children with ADHD tools to become aware of their distractibility and impulses, and helping them regulate themselves (Meppelink et al., 2024). Mindfulness training is originally developed to heighten awareness, improve concentration and reduce automatic responding (Kabat-Zinn, 2003). The MYmind training for children with ADHD and their parents teaches children with ADHD to focus and deepen their attention, and apply this skill in school, at home, and in their social life. Furthermore, children learn to become aware of thoughts, emotions, bodily sensations, and impulses rather than to react immediately, which can help them regulate their hyperactivity and impulsivity, and internalizing and externalizing behavior problems. Additionally, the MYmind training helps parents to cultivate a more accepting and non-reactive stance toward their child’s behavior, become mindful role models, help generalize mindfulness skills into the child’s daily life, lower their parenting stress and parental overreactivity, and if present, reduce their own ADHD symptoms (Bögels et al., 2021).

Studies have shown mindfulness to be effective for improving numerous mental conditions, including depression, anxiety, stress, insomnia, addiction, psychosis, and prosocial behaviors, and in different settings, including schools, workplaces, and in healthcare settings (D. Zhang et al., 2021). Furthermore, mindfulness meditation can improve certain attentional processes, including generalized attention, and its alerting networks and executive functioning (Sumantry & Stewart, 2021), and working memory (Whitfield et al., 2021), which could be beneficial especially for the ADHD population.

Systematic reviews and meta-analyses have found significant effects of mindfulness training in decreasing the severity of ADHD core symptoms in both adults (Oliva et al., 2021; Poissant et al., 2019), and children and adolescents with ADHD (Chimiklis et al., 2018; Evans et al., 2017; Lee et al., 2022; Oliva et al., 2021; Tan & Jones, 2024; J. Zhang et al., 2018). However, due to insufficient methodologically sound evidence to support the effectiveness, all authors conclude that no strong conclusions can be drawn.

Four recent randomized controlled trials (RCTs) support the MYmind family mindfulness training as an effective intervention in reducing ADHD symptoms in children and adolescents with ADHD (Meppelink et al., 2024; Siebelink et al., 2021; Valero et al., 2021; Wong et al., 2023).

Siebelink et al. (2021) compared MYmind in addition to Care-as-Usual (CAU) with CAU-only. In the short term they found that in the condition MYmind with CAU, significantly more children showed reliable improvement in executive functioning and ADHD symptoms significantly reduced according to parent and teacher ratings per protocol. Only parent-rated hyperactivity/impulsivity remained significantly reduced at 6 months follow-up. Parents improved on their own ADHD symptoms, well-being, and mindful parenting in the short term. In the longer term, some parental improvements remained (ADHD symptoms, mindful parenting), some additional significant effects appeared (other psychological symptoms, self-compassion) and others disappeared/remained non-significant.

Valero et al. (2021) randomized children with ADHD and their parents to MYmind or wait-list. In the short term no significant effects were found concerning the children. In the longer term (at 6 months follow-up) significant improvements in children’s inattention, executive functions, learning problems, aggression, and peer relations were reported in the MYmind condition. In the short term parents reported a decrease in parenting stress and improvement in parenting styles. Concerning the parenting variables only a significant reduction of parental overreactivity was maintained in the MYmind condition at follow-up.

Wong et al. (2023) compared the effects of MYmind versus Cognitive Behavior therapy (CBT) in a RCT with pretest, posttest, 3-months, and 6-months follow-up. Both MYmind and CBT significantly improved on the primary outcome measure of children’s attention (as measured with and objective attention test) and most secondary outcome measures (child behavior and parental measures), with no differences between conditions at any time points.

Meppelink et al. (2024) conducted a RCT comparing the effects of MYmind versus medication on attention problems and hyperactivity/impulsivity in children with ADHD and the current study uses their outcomes. Families were assessed at pretest, 2, 4, and 10 months. Between the 4- and 10-months assessment families no longer had to adhere to their original treatment. In the short term (at 2- and 4-months), all children improved substantially on ADHD-questionnaires, but medication was more effective than MYmind on multi-informant questionnaires. On objective (neuropsychological and blind observer) measures both conditions improved and no differences between MYmind and medication were observed. Differences between treatments diminished at 4 months even though in the medication condition treatment was continued after 2 months while the mindfulness training was already finished at 2 months. Differences between the two conditions disappeared at 10 months, but note that in the long-term, children from the mindfulness arm started taking medication and vice versa, so it is not sure to what extent the long-term results are caused by mindfulness, medication, or its combination. In addition to the RCT, Meppelink et al. (2024) analyzed a preference trial, where families received the treatment of their choice instead of being randomized. The preference trial demonstrated similar results, supporting the robustness of findings.

Aside from being a potentially effective treatment for children with ADHD, it is important to know whether mindfulness training is cost-effective; and thus not only increases the health benefits but also offer value for money. As far as we know, only one study assessed cost-effectiveness of mindfulness training in individuals with ADHD, who were of adult age. In this study, mindfulness training (MBSR) was added to treatment as usual (TAU) and compared with TAU (Janssen et al., 2019). TAU consisted of pharmacotherapy and/or psychosocial treatment such as psychoeducation and skills training. Effects were assessed on quality of adjusted life years (QALYs) and in response rate concerning the reduction of ADHD symptoms after treatment. Mindfulness training was found to be more costly and effective, particularly in terms of reduction of ADHD symptoms. Mindfulness training was found to be cost-effective if the threshold exceeded €30,000 per QALY and €1,000 per responder (Janssen et al., 2019).

The present study is the first to evaluate the cost-effectiveness of family mindfulness training (MYmind) in children and adolescents with ADHD, directly compared with methylphenidate, the recommended first-line and most consistently supported treatment for reducing core ADHD symptoms. It builds on the primary outcome study by Meppelink et al. (2024), which assessed the effects of both treatments on ADHD symptoms but did not examine ADHD-related costs. Because ADHD affects multiple areas of daily functioning, including healthcare use, educational support, and parental work productivity, its economic consequences extend beyond the healthcare sector alone. Evaluating cost-effectiveness solely from a healthcare (payer) perspective may therefore underestimate the broader economic impact of treatment. For this reason, the present study primarily adopted a societal perspective, incorporating all relevant ADHD-related costs. Primary outcome measures included inattention and quality-adjusted life years (QALYs). Secondary analyses examined additional outcomes, including hyperactivity/impulsivity, internalizing and externalizing problems. Furthermore, secondary analyses explored the healthcare (payer) perspective, shorter time horizons, the use of an alternative inattention questionnaire, per protocol analysis, and exclusion of participants who crossed over to the other treatment (i.e., received medication after MYmind or vice versa).

## Methods

### Design

This study used data from a multicenter preregistered RCT (Meppelink et al., 2016), designed as a real-life study comparing the (cost-)effectiveness of two treatment approaches for childhood ADHD: an established, evidence-based standard treatment (methylphenidate) and a relatively new but promising intervention (family mindfulness training). Recruitment took place via referrals from general practitioners or mental health care professionals as well as through (social) media. Families who expressed interest were screened for eligibility by a therapist. If inclusion criteria were met, detailed information about the study was provided and written consent was obtained.

Participants were randomized to either a mindfulness arm or a methylphenidate arm and completed the pre-test assessment. Then they started their 2-months treatment, after which they completed the second assessment. Participants in the methylphenidate arm received an additional 2 months of continued methylphenidate treatment, while participants in the mindfulness arm received a booster session at 4 months (i.e., 2 months after completing mindfulness training), followed by the 4-month assessment.

During the following 6 months, participants were free to choose their own course of treatment, including crossover to the other intervention. This approach was chosen for ethical and clinical reasons, as restricting access to evidence-based care (e.g., medication) for children who did not sufficiently benefit from the allocated treatment would not reflect routine clinical practice. The final assessment took place after this follow-up period, at 10 months.

### Participants

The sample for randomization comprised 98 families, with 51 allocated to MYmind and 47 to medication. Several participants dropped out or were excluded after randomization, primarily because they declined the allocated treatment or because the treatment did not start fast enough (see Figure 1). Ultimately, 88 children (mean age 11.27, 70% boys) received the allocated treatment; mindfulness (*n* = 46) or medication (*n* = 42). In total, 166 parents participated (mean age 45.42, 47% fathers) in the RCT; 64% had a university or college degree, 20% completed vocational education, 15% completed secondary school or less, and 1% of the educational level data was missing.

**Figure 1.**
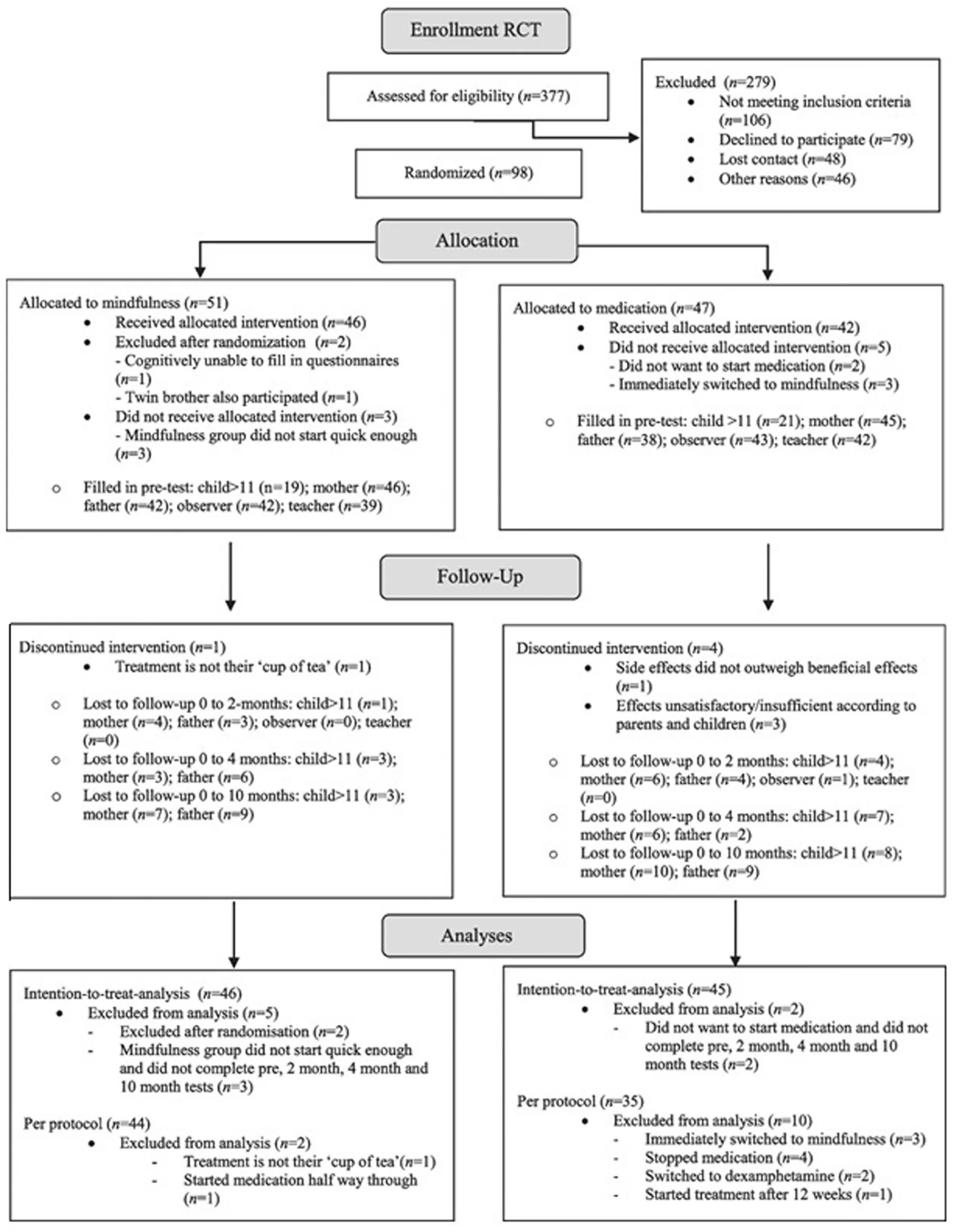
CONSORT flowchart randomized controlled trial. *Note.* This figure was published previously in Meppelink et al. (2024).

Inclusion criteria were: (1) an ADHD diagnosis, (2) an age between 9 and 18 years, (3) an IQ over 80 as measured by an abbreviated version of the Wechsler Intelligence Scale for Children – Third edition (WISC-III-NL; Kort et al., 2002), and (4) at least one parent willing to participate in the mindfulness training. Exclusion criteria were: (1) insufficient comprehension of the Dutch language by the child or parent(s), (2) psychosis, schizophrenia, or untreated Post Traumatic Stress Disorder (PTSD), (3) co-morbid behavior problems that were so severe, already during intake, that talking between the parent, child, and interviewer at intake was not possible, (4) methylphenidate use or mindfulness training during the last year, and (5) other, concurrent psychological interventions.

### Setting

To participate in the study, families needed a referral by a general practitioner or mental healthcare professional. The RCT was conducted in two outpatient child and family mental healthcare centers in the Netherlands, one more urban and one more rurally located.

### Interventions

The MYmind protocol (Bögels, 2020) was used in the mindfulness condition, in which children and adolescents followed eight weekly 1.5-hr group sessions in groups of approximately six children or nine adolescents. In parallel, parents followed an equally intensive mindful parenting program, aimed at cultivating mindfulness in parenting, including reducing parental stress and overreactivity, repairing parent-child conflicts, awareness of own limits, acceptance of the child’s ADHD, and self-compassion. Core themes of the child program included beginners mind, attention to the breath, coming home to the body, using mindfulness to deal with distractors, to regulate stress and impulsivity, and enhance autonomy. Joint parent–child components were included during parts of sessions 1 and 5, as well as during session 8 and the follow-up session, to facilitate shared learning and integration of skills. Both children and parents received workbooks and were asked to engage in daily home practice using guided meditation exercises. Two months after completion of the training, families attended a 1.5-hr booster session and were encouraged to continue practicing according to an individualized meditation plan.

Treatment integrity of MYmind was assessed using the MYmind Treatment Adherence and Competence Scale (MYmind-TACS; Ridderinkhof et al., 2017). A substantial proportion of sessions were videotaped (66% of child sessions and 70% of parent sessions). Interrater reliability was excellent (ICC range = .80–.88). Trainers showed high adherence to the protocol (85.8% for child sessions and 91.6% for parent sessions delivered fully as intended), and competence ratings were high (*M* = 4.29–4.60 on a 5-point scale).

In the medication condition, children received short-acting methylphenidate prescribed and monitored by a child psychiatrist according to the Dutch Multidisciplinary Guidelines for ADHD (Trimbos-instituut, 2005). After the initial consultation, children started with three daily doses of 2.5 or 5 mg methylphenidate, administered 7 days/week. Medication was titrated individually based on clinical response and side effects. Families were contacted weekly by the psychiatrist until optimal titration was achieved, and follow-up consultations took place approximately every four weeks. If methylphenidate was insufficiently effective or side effects outweighed benefits, dosage adjustments were made, medication type was changed, or treatment was discontinued when necessary.

### Measures

#### Costs

Using a retrospective cost questionnaire based on the one used by Van Steensel et al. (2013), parents were asked to report over the preceding 2 months on all resource usage related to the child’s ADHD (healthcare usage, hospital visits, medication usage, help or aid from professionals or family and friends), including loss of daily activities and (paid) work. The cost questionnaires were administered at all four assessment time points and an extra two time points during the 6-months follow-up period that is, at 0, 2, 4, 6, 8, and 10 months. Costs were calculated by multiplying the resources used with their respective unit price. All societal costs, including both healthcare and non-health care costs – were taken into account. Societal costs provide the most comprehensive assessment of the economic impact of treatments (Neumann et al., 2016). However, healthcare costs are of particular relevance to payers such as insurers; therefore, separate analyses were conducted from a healthcare perspective only. Most unit prices were obtained from the Dutch Guideline for Cost-Research (Hakkaart-van Roijen et al., 2015). Costs for general practitioners outside working hours were obtained from the Dutch Healthcare Authority; costs of students requiring extra attention were obtained from the CPB Netherlands Bureau for Economics Policy Analysis; costs of a Personal Care Budget (further referred to by the Dutch abbreviation: PGB) and costs of education were obtained from the Dutch government. Medication costs were mostly derived from the Dutch “Pharmaceutical Compass” website; for more unusual medication or medication sold over-the-counter that where not covered by the “Pharmaceutical Compass” website, the websites of regular drugstores were used. To calculate the loss of daily activities of children, the price unit of school absence was used as a shadow price; an official price unit was not available because daily activities of children are hardly quantifiable. We calculated intervention costs for participants in both conditions separately. Concerning the mindfulness condition the hours of the training (20 hr including preparations) were multiplied with the hourly rate of three psychologists (two for the child group and one for the parent group). Subsequently, the costs were divided by the average number of children (six concerning the younger children, nine concerning adolescents), leading to 667.90 euro for the child groups and 445.27 euro for the adolescent groups. In the medication condition the number of consults were multiplied with the rate of a psychiatrist and the dose of the methylphenidate usage with its unit price. The costs were indexed using the consumer price index to 2018. See Table 1 for a unit costs list including healthcare and non-healthcare costs.

**Table 1.** Costs for Participants in MYmind and Medication (in 2018 Euro).

Resource	Unit Price	MYmind (*n* = 46)		Medication (*n* = 42)		Mean incremental cost (95% CI)
		*M*	*SD*	*M*	*SD*	
*Healthcare costs*						
General practitioner (GP)						
*Telephone*	17.74 p/c	2.98	6.39	2.42	7.01	
*Visit*	34.44 p/c	29.96	39.49	32.87	40.56	
GP outside working hours						
*Telephone*	25 p/c	1.13	5.15	0.71	3.85	
*Visit*	107.10 p/c	5.89	21.94	13.94	48.69	
Paramedic care						
*Physiotherapy*	33.94 p/c	26.70	86.9	29.09	72.79	
*Other paramedic care*	33.94 p/c	26.15	66.04	22.55	67.41	
*Mental health care*						
Psychologist/Educationalist						
*Telephone*	34.73 p/c	22.48	51.08	5.37	14.05	
*Visit*	66.79 p/c	885.87	275.39	616.33	520.02	
Psychiatrist						
*Telephone*	51.25 p/c	32.84	59.53	57.53	69.24	
*Visit*	98.56 p/c	157.54	136.78	420.23	219.40	
*IPG*	159.27 p/c	83.62	336.62	26.83	67.38	
PGB^a^	Variable	236.75	1,419.45	80.95	400.49	
Medication	Variable	47.41	37.64	112.63	83.13	
*Subtotal*		1,580.75	1,641.11	1,414.39	660.60	166 [−373, 706]
*Non-healthcare costs*						
Child’s school absence	8.20 p/h	225.67	233.56	242.15	416.44	
Consultation school	Variable	290.89	345.57	163.18	195.69	
Loss of daily activities child	8.20 p/h	100.70	166.74	108.01	295.79	
Loss of paid work mother	32.98 p/h	152.59	458.16	118.84	394.15	
Loss of paid work father	39.55 p/h	145.05	410.57	39.19	142.30	
Help from family/friends	14.61 p/h	51.16	118.11	179.42	770.30	
Other (out of pocket) expenses	Variable	239.42	416.35	242.70	325.47	
*Subtotal*		1,168.38	1,042.10	1,060.23	1,357.37	108 [−402, 618]
Total costs		2,749.12	2,328.71	2,474.62	1,696.29	275 [−596, 1,145]
T1-T2 (0–2 months)		1,084.19	841.77	512.83	340.92	571 [294, 841]
T2-T3 (2–4 months)		327.02	585.03	493.20	403.60	−166 [−381, 49]
T3-T4 (4–10 months)		1,316.49	1,276.84	1,475.63	1,396.17	−159 [−726, 407]

*Note.* IPG = intensive pedagogical family guidance; p/c = per contact; p/h = per hour; p/d = per day; p/2mnd = per 2 months.

a In the Netherlands one can apply for a personal budget (PGB) for a child with a DSM-V diagnosis to buy additional help, which varies from 1,577 to 45,581 per year.

#### Effects

To assess the effects of both interventions, questionnaires were administered during all four assessment times. ADHD symptoms are measured by two subscales (Inattention and Hyperactivity/Impulsivity) of the Dutch parent version of the Disruptive Behavior Disorder Rating Scale (DBDRS), which has adequate reliability, convergent, and discriminant validity (Oosterlaan et al., 2000). Both subscales consist of nine items using a 4-point Likert scale. A score of 15 or higher is considered a (sub)clinical score.

Furthermore, different aspects of children’s behavioral and emotional problems were assessed with the Child Behavior Checklist (CBCL; T. Achenbach, 1991). The CBCL is a questionnaire of 113 items using a 3-point Likert scale, with high reliability and validity (T. M. Achenbach & Rescorla, 2001). The subscales Attention problems, Internalizing problems and Externalizing problems were used. A *T*-score of 60 or higher was considered a (sub)clinical score concerning Internalizing and Externalizing problems, and a *T*-score of 65 or higher was considered a (sub)clinical score concerning Attention problems (T. M. Achenbach & Rescorla, 2001).

Children’s health-related quality of life was measured with the EuroQol EQ-5D-Y-3L (Devlin & Brooks, 2017). The questionnaire has five different dimensions (Mobility, Self-care, Usual activities, Pain/Discomfort, and Anxiety/Depression), on which parents were asked to indicate their child’s health state by picking the most appropriate statement out of three. For each dimension, a utility score can be calculated by attaching preference weights to each statement (Weinstein et al., 2009). A utility score is measured on a cardinal scale ranging from 0 to 1, where 0 indicates death and 1 indicates the best imaginable health state. This utility score can then be used to calculate QALY which is a summary measure for the time spent in a certain health state; one QALY is equal to 1 year in full health (Whitehead & Ali, 2010). The questionnaire has been widely used and has good psychometric properties (Noyes & Edwards, 2011).

### Data and Analyses

All allocated participants were included, regardless of treatment adherence or completion of all measurements. In the study protocol, the Youth Self-report (YSR; T. Achenbach, 1991), the EQ-5D-Y-3L Self-report (Devlin & Brooks, 2017), and the adult Self-report Questionnaire for Inattention and Hyperactivity (SQIH; Kooij et al., 2004) were also described as outcome measures in the cost-effectiveness analyses. However, due to a large number of missing data concerning the YSR, partly because only adolescents from 12 years and older could fill this in, and the EQ-5D-Y-3L Self-report (39% and 22% of data was missing respectively) we excluded these measures. Furthermore, because relatively few parents (six mothers and six fathers) met the (sub)clinical threshold of ADHD at pretest on the SQIH, we also excluded these measures.

MCAR analyses showed that data was missing at random and incomplete data was imputed using SPSS missing value analysis. Chi-square analyses were used to examine differences between conditions with respect to the percentage of children being free from (sub)clinical attention problems, hyperactivity/impulsivity, internalizing and externalizing problems. *T*-tests were conducted to examine differences in QALYs, and, if not normally distributed, also Mann-Whitney *U* tests were conducted for verification of the results. Cost-differences were examined with bootstrap analyses (with replacement). In bootstrapping, a large amount of resamples (1,000 in this study) are drawn from the original sample to estimate a statistic’s sampling distribution (Mooney & Duval, 1993).

To evaluate the (incremental) cost-effectiveness of one intervention over the other, incremental cost-effectiveness ratio’s (ICERs) were calculated. The ICER is expressed as the incremental change in costs divided by the incremental effects. In the primary analyses, ICERs were expressed as (1) the cost per child being free of (sub)clinical attention problems as measured with the DBDRS, and (2) the cost per QALY. Because the study did not cover a full year but 10 months, the highest possible value of the QALY is 0.83.

Bootstrapping was used to construct confidence intervals around the ICER, which is a suitable method to quantify the uncertainty around the ICER that does not depend on the usual parametric assumptions of the sampling distribution (Briggs et al., 1997; Campbell & Torgerson, 1999). The uncertainty around the ratio can be presented graphically in a cost-effectiveness plane (O’Brien & Briggs, 2002); the differences in effects are plotted on the *x*-axis and the differences in costs on the *y*-axis. To estimate the probability that mindfulness is cost-effective compared to methylphenidate use, cost-effectiveness acceptability curves (CEAC’s) were constructed. The CEAC’s indicate the probability that mindfulness is cost-effective compared with methylphenidate for a range of ceiling ratios. These are the maximum costs that one (society) should be willing to pay for a child free of certain (sub)clinical behavioral or emotional problems/QALY (Fenwick et al., 2004).

Primary analyses were based on parent reported (sub)clinical attention symptom scores on the DBDRS and the parent report of the EuroQol EQ-5D-3L, while considering a societal perspective (including all healthcare and non-healthcare costs related to the child). To test the robustness of results, and having a better understanding and interpretation of the results, secondary analyses were run where outcome measures and perspectives were varied: (1) taking the scale hyperactivity/impulsivity of the DBDRS as an outcome measure, (2) taking the CBCL scale Attention problems as an outcome measure, (3) taking the CBCL Internalizing scale as an outcome measure, (4) taking the CBCL Externalizing scale as an outcome measure, (5) taking a healthcare approach (instead of a societal approach), (6) taking shorter time horizons (2 and 4 months instead of 10 months), (7) per protocol analysis (participation in ≥4 MYmind sessions or continuation of methylphenidate between 4 and 10 months), (8) excluding children that followed medication after mindfulness training and vice versa.

## Results

### Description of MYmind and Medication Condition

No baseline differences were observed between the two conditions in terms of gender, X^2^(1, *n* = 88) = 0.04; *p* = .85, or age, *t*(86) = −0.44, *p* = .66. Similarly, there were no differences in baseline QALYs, *t*(86) = 0.58, *p* = .57, or in (sub)clinical attention problems measured with the Inattention scale of the DBDRS, X^2^(1, *n* = 88) = 0.55. Bootstrap analyses further indicated no differences between MYmind and medication in baseline total costs, *M*_incremental costs_ = −€14; 95% CI [−€292, 237].

Finally, no significant baseline differences were found between the conditions concerning the presence of (sub)clinical problems on the secondary outcome measures; Hyperactivity/impulsivity (DBDRS), X^2^(1, *n* = 88) = 0.67; *p* = .41, Attention problems (CBCL), X^2^(1, *n* = 88) = 1.76; *p* = .19, Internalizing problems (CBCL), X^2^(1, *n* = 88) = 2.065; *p* = .15, and Externalizing problems (CBCL), X^2^(1, *n* = 88) = 0.59; *p* = .44 (Figure 2).

**Figure 2.**
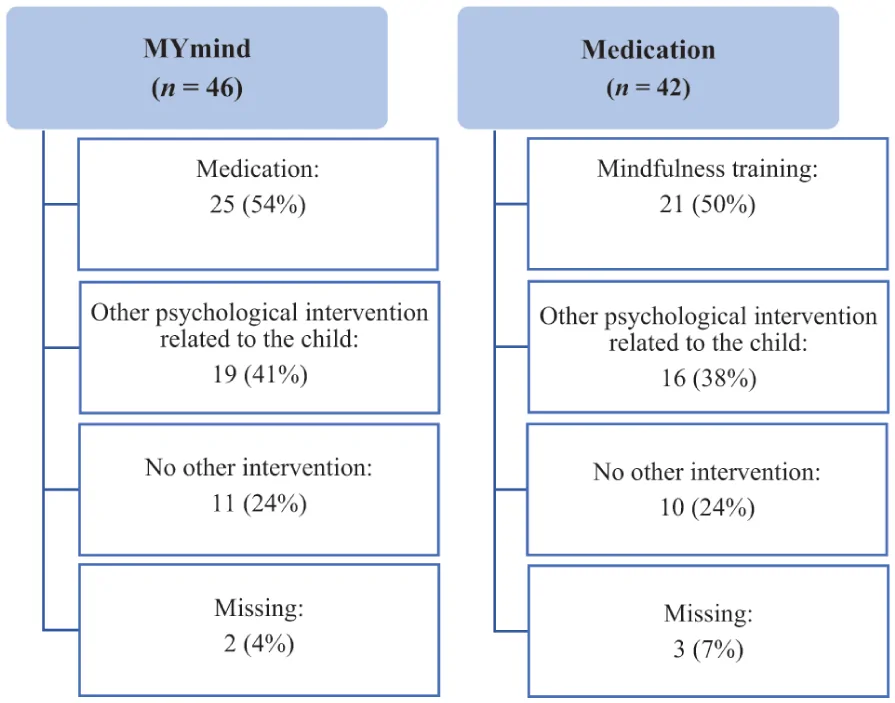
Flowchart of children’s treatments during follow-up (4–10 months after pretest).

### Costs and Effects

There were no statistical differences between the two conditions in terms of total costs, healthcare costs, or non-healthcare costs, on all different time intervals except for the intervention period interval (0–2 months) in which costs were higher in the MYmind condition than in the medication condition (cost difference = €571; see Table 1).

With regard to the outcome measures, at 2 and 4 months the percentage of children with (sub)clinical attention problems and hyperactivity/impulsivity problems was significantly lower in the medication condition compared to the MYmind condition, as measured with the DBDRS. At 10 months no significant differences were found between MYmind and medication. Concerning QALY and all CBCL subscales, no significant differences were found between MYmind and medication on all moments in time. See Table 2 for a full overview of the outcome measures.

**Table 2.** Comparisons Between MYmind and Medication on the Outcome Measures.

Variable	MYmind (*n* = 46)	Medication (*n* = 42)	Test statistic	*p*-value
Normal scores on DBDRS inattention	*n* (%)	*n* (%)	X^2^	
2 months	15 (33)	31 (74)	14.94	<.001
4 months	17 (37)	28 (67)	7.76	.006
10 months	23 (50)	23 (55)	0.20	.68
QALY	*M* (*SD*)	*M* (*SD*)	*T*	
2 months	0.14 (0.04)	0.14 (0.04)	0.38	.70
4 months	0.31 (0.03)	0.30 (0.05)	0.85	.40
10 months	0.76 (0.12)	0.73 (0.14)	0.85	.40
Normal scores on DBDRS hyperactivity/impulsivity	*n* (%)	*n* (%)	X^2^	
2 months	29 (63)	38 (90)	9.09	.003
4 months	33 (72)	38 (90)	4.95	.032
10 months	38 (83)	38 (90)	1.15	.36
Normal scores on CBCL inattention	*n* (%)	*n* (%)	X^2^	
2 months	27 (59)	31 (74)	2.23	.18
4 months	31 (67)	32 (76)	0.84	.48
10 months	30 (65)	32 (76)	1.27	.35
Normal scores on CBCL internalizing problems	*n* (%)	*n* (%)	X^2^	
2 months	37 (80)	34 (81)	0.004	1.00
4 months	38 (83)	37 (88)	0.53	.56
10 months	40 (87)	35 (83)	0.23	.77
Normal scores on CBCL externalizing problems	*n* (%)	*n* (%)	X^2^	
2 months	38 (83)	34 (81)	0.04	1.00
4 months	34 (74)	35 (83)	1.15	.31
10 months	39 (85)	38 (90)	0.65	.53

*Note.* DBDRS = disruptive behavior disorders rating scale; CBCL = Child Behavior Checklist; QALY = Quality Adjusted Life Years.

### Cost-Effectiveness Analyses

The ICER based on the costs per child free of (sub)clinical attention problems, as measured by the primary outcome measure DBDRS, demonstrated that MYmind was inferior to medication at 10 months; the effectiveness of MYmind was 0.11 lower and the costs were 246 euro higher (Table 3). The cost-effectiveness plane (Figure 3) demonstrated that most of the point estimates (86%) were found on the left side of the vertical axis, indicating a high probability that MYmind is less effective than medication. In addition, most point estimates (73%) were found above the horizontal axis, indicating a rather high probability that MYmind costs more than medication. More specifically, 21% of the ICERs were falling in the South-West quadrant (MYmind is less effective, and costs are lower), 65% in the North-West quadrant (MYmind is less effective, and costs are higher), 8% in the North-East quadrant (MYmind is more effective, and costs are higher), and 6% in the South-East quadrant (MYmind is more effective, and costs are lower; Table 3). The cost-effectiveness acceptability curve (Table 3) demonstrated that MYmind has a probability of 27% of being cost-effective when the willingness to pay for one child without (sub)clinical attention problems equals zero. This probability decreases with higher willingness to pay thresholds.

**Table 3.** Effects, Costs, and Cost-Effectiveness Analyses for MYmind Versus Medication.

Analysis		MYmind		Medication			% ICERs falling into the four quadrants of the CE-plane				Probability that MYmind is cost-effective at willingness to pay of €		
*Primary*	Measures	Costs	Effects	Costs	Effects	ICER	SW	NW	NE	SE	0 (%)	10,000 (%)	20,000 (%)
	Inattention	2,728	0.65	2,482	0.76	Inferior	21	65	8	6	27	12	12
	QALY	2,728	0.79	2,482	0.78	€38.089	3	26	44	27	30	38	43
*Secondary*													
2 months	Inattention	1,084	0.33	513	0.74	Inferior	1	99	0	0	1	0	0
	QALY	1,084	0.16	513	0.15	€223.456	0	19	82	0	0	0	0
4 months	Inattention	1,411	0.37	1,006	0.67	Inferior	2	98	0	0	2	0	0
	QALY	1,411	0.31	1,006	0.31	€106.072	0	20	78	3	3	4	8
10 months	Hyp/imp	2,728	0.83	2,482	0.90	Inferior	24	61	9	6	30	13	14
	CBCL Att	2,728	0.65	2,482	0.76	Inferior	21	65	8	6	27	12	13
	CBCL Int	2,728	0.87	2,482	0.83	€6.791	3	30	42	25	28	53	60
	CBCL Ext	2,728	0.85	2,482	0.90	Inferior	19	61	12	8	27	18	19
Healthcare pers.	Inattention	1,559	0.65	1,421	0.76	Inferior	29	58	7	6	34	10	11
	QALY	1,559	0.79	1,421	0.78	€21.347	7	22	45	26	33	45	52
Per protocol	Inattention	2,734	0.50	2,218	0.60	Inferior	7	76	16	2	8	11	15
	QALY	2,734	0.79	2,218	0.79	Inferior	3	65	27	5	8	9	11
Only MYmind/only MED	Inattention	2,095	0.48	2,486	0.48	Dominant	37	9	10	44	81	60	55
	QALY	2,095	0.79	2,486	0.78	Dominant	11	7	13	70	81	84	86

*Note*. All analysis were conducted at 10 months unless specified otherwise in the first column. Inattention and Hyperactivity/impulsivity as described in the second column was measured with the Disruptive Behavior Disorder Rating Scale. ICER = incremental cost-effectiveness ratio; CE-plane = cost-effectiveness plane; NE = North-East (MT > effect and >costs than MED); NW = North–West (MT < effect and >costs than MED); SE = South-East (MT > effect and <costs than MED); SW = South-West (MT < effect and <costs than MED); QALY = quality adjusted life year; Hyp/imp = Hyperactivity/impulsivity; CBCL = Child Behavior Checklist; Att = Attention problems; Int = Internalizing problems; Ext = externalizing problems; Healthcare pers. = Healthcare perspective.

**Figure 3.**
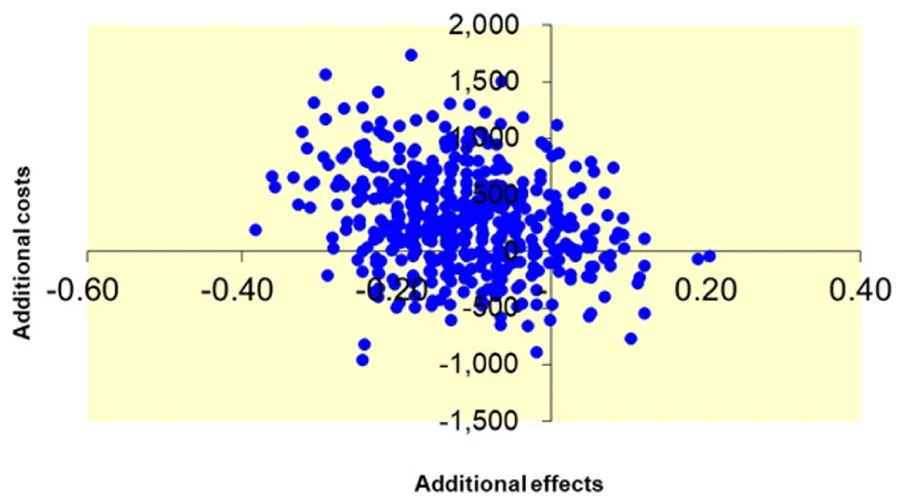
Cost-effectiveness plane for MYmind versus medication concerning attention problems (as measured with the DBDRS). *Note.* Costs are represented as total costs between 0 and 10 months assessment; effects are represented as percentage free of (sub)clinical attention problems at 10 months.

The ICER based on the costs per QALY demonstrated that MYmind was more effective and more expensive at 10 months; the effect of MYmind was 0.01 higher and the costs were 246 euro higher (Table 3). The cost-effectiveness plane (Figure 4) demonstrated that most of the point estimates (71%) were found on the right side of the vertical axis, indicating a rather high probability that MYmind is more effective than medication. Furthermore, most point estimates (70%) were found above the horizontal axis, indicating a rather high probability that MYmind costs more than medication. More specifically, 3% of the ICERs were falling in the South-West quadrant (MYmind is less effective, and costs are lower), 26% in the North-West quadrant (MYmind is less effective, and costs are higher), 44% in the North-East quadrant (MYmind is more effective, and costs are higher), and 27% in the South-East quadrant (MYmind is more effective, and costs are lower; Table 3). The cost-effectiveness acceptability curve (Table 3) demonstrated that MYmind has a probability of 30% of being cost-effective when the willingness to pay for a QALY equals zero. This probability increases as the willingness to pay increases.

**Figure 4.**
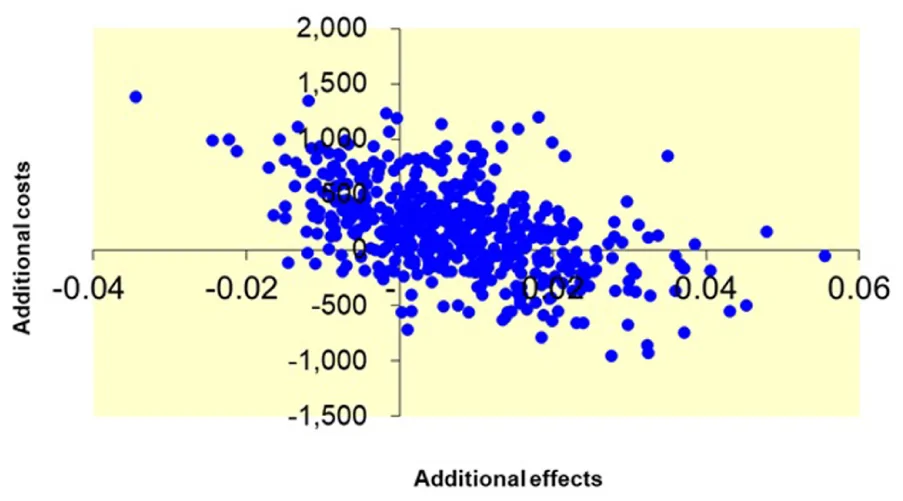
Cost-effectiveness plane for MYmind versus medication concerning quality adjusted life years. *Note.* Costs are represented as total costs between 0 and 10 months assessment; effects are represented as quality adjusted life years.

The results of the secondary cost-effectiveness analyses are also displayed in Table 3 and show similar results. Regarding CBCL Attention problems and Externalizing problems, MYmind is mostly inferior to medication and, with regards to QALY’s and CBCL Internalizing problems, MYmind is generally more effective but also more expensive. Similarly, from a healthcare (payer) perspective, MYmind is inferior to medication concerning DBDRS Inattention, while it remains more effective but also more expensive in terms of QALY’s. Only in the per protocol analyses MYmind is inferior to medication also concerning QALY’s. Probability rates of MYmind being cost-effective compared with medication range from 0% to 34% at a willingness to pay equal to zero (Table 3). There is one noteworthy exception; when excluding children that took medication after following mindfulness training and vice versa (thus selecting the children who did not follow the other treatment after the 4 months period, allowing for a “pure” comparison of the assigned treatments); on both outcome measures (Inattention measured with the DBDRS and QALY’s) MYmind was dominant to medication. Concerning Inattention, 54% of the point estimates were found on the right side of the vertical axis, indicating a rather equal probability that MYmind is more effective than medication in this subgroup. Furthermore, most point estimates (81%) were found below the horizontal axis, indicating a high probability that MYmind costs less than medication. Regarding QALYs, 83% of the point estimates were found on the right side of the vertical axis, indicating a high probability that MYmind is more effective than medication in this subgroup. Additionally, most point estimates (81%) were found below the horizontal axis, indicating a high probability that MYmind costs less than medication. Bootstrapped ICERs in this subgroup demonstrated that the probability of MYmind to be cost-effective is 81% for both a child free of (sub)clinical attention problems and for a QALY when the willingness to pay equals zero. See Supplemental Figures 1 and 2.

## Discussion

This was the first study to compare mindfulness training (MYmind) versus medication in children and adolescents with ADHD, taking into account both effects and costs. Cost-effectiveness analyses demonstrated MYmind being mostly inferior when considering ADHD core symptoms and MYmind having rather high chances of being more effective and more expensive when considering quality of life (QALY). These findings were consistent from both a societal and healthcare (payer) perspective. The chances of MYmind being cost-effective over medication are the lowest in the short and middle term and increase somewhat in the long term (but chances to be cost-effective were still low), with the highest chance of MYmind being cost-effective over medication found for the scenario where families that participated in both treatments were excluded.

Primary analyses showed that with respect to the costs per child free of (sub)clinical attention problems in the long term, MYmind was inferior to medication. The probability that MYmind is cost-effective is 27% when the willingness to pay for one child without (sub)clinical attention problems equals zero. This probability decreases with higher willingness to pay thresholds. This finding is in line with the small number of studies that show low chances of psychosocial treatments to be cost-effective compared to medication in the ADHD population (Dijk et al., 2021). With respect to the QALYs, the ICER demonstrated that MYmind had rather high chances of being more effective but also to be more expensive in comparison with medication. The probability that MYmind is cost-effective ranges from 30% to 43%, depending on the amount of willingness to pay (range = €0–€20.000).

Secondary analyses mostly showed similar results; MYmind is mostly inferior to medication, however, with regards to QALY’s and internalizing problems MYmind has rather high chances of being more effective but also more expensive in comparison with medication. Probability rates of MYmind being cost-effective compared with medication ranging from 0% to 34% at a willingness to pay equal to zero.

One noteworthy exception was when excluding participants that took medication after following mindfulness training and vice versa; on both outcome measures (Inattention and QALY’s) MYmind was dominant to medication. Bootstrapped ICERs in this subgroup demonstrated that the probability of MYmind to be cost-effective is 81% for both a child free of (sub)clinical attention problems and for a QALY when the willingness to pay equals zero. One reason for MYmind being the dominant intervention in this scenario is that costs for medication and doctors’ visits continue, while mindfulness can be applied by families themselves for free once the training is finished. Consequently, when assessing long-term costs of MYmind versus medication while removing families that participated in both treatments, results indicate that MYmind is actually less expensive than medication. Concerning the effects we see that the percentage of children with (sub)clinical attention problems in MYmind or medication are similar in this subgroup in the long term. This is interesting, considering that in the total group in the short and middle term there were significantly more children with (sub)clinical attention problems in the MYmind condition compared to the medication condition. Post-hoc analyses showed that also in this specific subgroup significantly more children had (sub)clinical attention problems in MYmind in comparison with medication with regards to the short term (X^2^(1, *n* = 42) = 6.11; *p* = .02), but in the middle and longer term differences resolved. One possible explanation is that medication effects may decline due to adherence issues or adverse effects (Taylor, 2019), whereas mindfulness skills may require longer implementation time to consolidate and exert their full impact. Importantly, these findings are based on smaller subgroups (21 participants per condition) and are no longer randomized. Therefore, it is likely that these subgroups consist of participants for whom the assigned treatment was effective, and consequently, they did not seek the alternative treatment after the first four months of the trial. Therefore, results should be interpreted with caution. While these participants did not cross over to the alternative treatment, they did engage in other forms of care; however, post-hoc analysis indicated that both subgroups received the same number of additional interventions during follow-up, making it unlikely that these factors alone account for the observed patterns. As this secondary cost-effectiveness analysis excluding the crossover participants allows for a long-term comparison of the “clean” effects of mindfulness versus medication, it can be cautiously concluded that there exists a substantial subgroup for which mindfulness is cost-effective.

Although MYmind compared to medication was not found to be cost-effective, several considerations need to be made. First, the (limited) time horizon needs to be taken into account. Considering that medication shows increased costs and possibly reduced effects over time, in combination with a relatively large group that discontinues medication treatment (10% vs. 2% that discontinued MYmind in this study), medication might not be the ideal or a sufficient option for some children with ADHD in the long term. Second, medication and psychosocial interventions may differ in their effects across outcome domains. While medication generally shows stronger effects on core ADHD symptom reduction, psychosocial interventions such as MYmind may have relatively greater impact on functional and behavioral impairments. This distinction is relevant for interpreting the present findings, as the primary outcomes in this study focused largely on symptom reduction, which may have favored medication as the comparator in the cost-effectiveness analyses. Third, another critique concerning medication as an external agent, is that families do not receive tools themselves on how to deal with the ADHD symptoms. MYmind does provide children and parents with tools and shows significant – although more modest – positive effects on the core ADHD symptoms measures (see also Meppelink et al., 2024) and positive effects on comorbid (internalizing and externalizing) problems (Kosterman Zoller et al., 2025a). Finally, family-based MYmind is far more beneficial for parents of children with ADHD compared to medication. Mindfulness meditation helps them, among other things, to reduce parental stress, increase self-compassion, and improve different aspects of psychological well-being (Kosterman Zoller et al., 2025b). For these reasons, MYmind might still be considered a valid treatment option for children with ADHD and their parents.

Strengths of this study are that it concerns a preregistered, randomized controlled design, and that detailed information about costs – including non-healthcare costs – were obtained using cost questionnaires completed by participants at different points in time. Limitations of the study were that only parent reports were included and that, since families had free choice of treatment in the long term, the 10-month measure does not represent “pure” effects of mindfulness versus medication. The fact that about three quarters of the families followed at least two treatments and about half of the participants followed both MYmind and medication, however, also highlights the need in these families for better treatment outcomes. A combination of treatments might be more effective and needed for long term healthy functioning in the ADHD population. This is line with the study of Janssen et al. (2019) where mindfulness training was found to be a cost-effective addition to treatment as usual in adults with ADHD. Lastly, the study was limited by its regional focus, the relatively high educational level of parents, and the low proportion of parents meeting criteria for ADHD, suggesting that the sample may not fully represent the broader ADHD population. Therefore, caution is warranted when generalizing these findings, and replication in more diverse and clinically complex populations is needed.

Future cost-effectiveness research could focus on comparing MYmind, medication, and a combination of both, including longer-term measurements (e.g., after 1–2 years) preferably with longer periods of time adhering only to the allocated treatment, to help understand what ADHD treatment options are most cost-effective in the long run. In addition, future studies could strengthen cost estimates by combining questionnaire-based resource use data with more objective sources, such as national healthcare registries, to provide a more precise assessment of costs related to the child’s ADHD. Comorbid internalizing and externalizing problems could be included as moderators, to gain understanding which treatment(s) would be advisable for who. It is also recommended to include parental outcomes in future cost-effectiveness research, considering ADHD in children is related with significantly higher parenting stress in parents (Theule et al., 2013), and to broaden outcomes beyond symptom reduction to functional impairment and daily life functioning. Another recommendation is to compare MYmind for children and adolescents (with a minimum involvement of the parents) with a Mindful Parenting training for parents, and a combination of both, to understand which options are most cost-effective.

To conclude, this study can be seen as a first step to shed some light on the cost-effectiveness of mindfulness training versus medication in children with ADHD. We hope that the present study will stimulate further investigation of this important area to understand which ADHD treatment options are valid alternatives for, or additions to, medication.

## Supplemental Material

sj-jpg-1-jad-10.1177_10870547261469623 – Supplemental material for Cost-Effectiveness of a Child and Parent Mindfulness-Based Training Versus Medication for Childhood ADHD Supplemental material, sj-jpg-1-jad-10.1177_10870547261469623 for Cost-Effectiveness of a Child and Parent Mindfulness-Based Training Versus Medication for Childhood ADHD by Rachel T. van der Meulen, Francisca J. A. van Steensel, Esther I. de Bruin, Wiebe Korf and Susan M. Bögels in Journal of Attention Disorders

sj-jpg-2-jad-10.1177_10870547261469623 – Supplemental material for Cost-Effectiveness of a Child and Parent Mindfulness-Based Training Versus Medication for Childhood ADHD Supplemental material, sj-jpg-2-jad-10.1177_10870547261469623 for Cost-Effectiveness of a Child and Parent Mindfulness-Based Training Versus Medication for Childhood ADHD by Rachel T. van der Meulen, Francisca J. A. van Steensel, Esther I. de Bruin, Wiebe Korf and Susan M. Bögels in Journal of Attention Disorders

## Data Availability

Data of this study are stored in the University’s repository.
